# Candidate-related factors associated with success in the Fellowship of the College of Diagnostic Radiologists South Africa Part 2 examination

**DOI:** 10.4102/sajr.v30i1.3459

**Published:** 2026-07-03

**Authors:** Thabokgone Muwezwa, Halvani Moodley, Anushka Ajith

**Affiliations:** 1 Department of Radiology, Faculty of Health Sciences, University of the Witwatersrand, Johannesburg, South Africa; 2 Teaching and Learning Unit, Faculty of Commerce, Law and Management, University of the Witwatersrand, Johannesburg, South Africa

**Keywords:** FC Rad Diag, CMSA, success rate, pass rate, examination, COVID-19

## Abstract

**Background:**

The examination of the Fellowship of the College of Diagnostic Radiologists South Africa (FC Rad Diag [SA]), conducted by the Colleges of Medicine of South Africa (CMSA), is the national exit examination for qualification as a radiology specialist. There is limited published evidence on South African pass rates and factors influencing success in the FC Rad Diag (SA) Part 2 examination.

**Objectives:**

This study aimed to determine the pass rate and factors associated with success in the FC Rad Diag (SA) Part 2 examination.

**Method:**

A descriptive quantitative study was conducted using CMSA examination data from 2015 to 2023. Descriptive and inferential analyses were used to assess associations between candidate characteristics and examination outcomes.

**Results:**

A total of 377 candidates sat in the examination, with an overall pass rate of 85.7% (323/377). Candidates who failed were older (median age 42 years old [37–47 years] vs. 35 years old [33–38 years]; *p* < 0.001). Higher pass rates were observed among candidates in registrar training posts compared with medical officers (80.2% [259/323] vs. 19.8% [64/323]; *p* < 0.001). Successful candidates had more recently completed their Bachelor of Medicine and Bachelor of Surgery (MBBCh /MBChB) (median 11 [9–13] vs. 14.5 [12–17] years; *p* < 0.001) and Part 1 examinations (4 years [3–5 years] vs 5 years [4–6 years]; *p* < 0.001). They also had fewer examination attempts (median 1 [1–2] vs. 3.5 [2–5]; *p* < 0.001).

**Conclusion:**

Success in the FC Rad Diag (SA) Part 2 examination is influenced by multiple candidate-related factors.

**Contribution:**

Targeted academic support programmes should focus on candidates at higher risk of failure, particularly those outside formal registrar posts and those with multiple examination attempts, to improve equity and overall specialist throughput nationally.

## Introduction

The examination of the Fellowship of the College of Diagnostic Radiologists South Africa (FC Rad Diag [SA]), conducted by the College of Radiologists (CR) of the Colleges of Medicine of South Africa (CMSA), is the single exit examination required to qualify as a radiology specialist across South Africa. A Master of Medicine (MMed), which is the research component, is also a prerequisite for registering as a specialist with the Health Professions Council of South Africa (HPCSA). The CMSA has been tasked to conduct a national examination that is independent, fair and efficient.^1^ The FC Rad Diag (SA) examination is closely modelled on the Fellowship of the Royal College of Radiologists (FRCR) in London with the aim to remain on par with international standards.^2^

The FC Rad Diag (SA) examination currently consists of two components: Part 1 (radiation physics a 3 h written paper and imaging anatomy, two or three spot tests with no oral component); and Part 2 (3 written papers, a rapid reporting session and long cases). Unlimited attempts are allowed for Part 2, but this must be achieved within 8 years of obtaining Part 1 or the candidate will be required to repeat the Part 1 examination.^3^ The CMSA entry requirements to the Part 2 examinations include completion of 36 months of training as a radiology registrar in an HPCSA-recognised post, passed or exempt from the Part 1 examination and completion of a performance portfolio or logbook with a satisfactory continuous assessment signed by the head of department.^3^ The preparedness for any examination by a candidate is multifactorial. This places the spotlight on what factors influence candidate performance and ultimately, success.

Failure rates in training programmes for specialist examinations are high across different specialist medical colleges.^4,5^ This has prompted research that has identified a range of potential reasons for failure, including trainee personal factors^6,7^ and examination preparation factors.^4,7^ Trainee personal factors include age, ethnicity, undergraduate degree training institution, and possession of a postgraduate degree.^6,7,8^ Examination preparation factors include the development of a structured study plan, the presence of support structures, and understanding the examination format.^4^

A study carried out in the United Kingdom (UK) looked at the success in the FRCR Part 2B (UK) examination from 2006 to 2010.^8^ Factors such as gender, ethnicity, number of attempts at the examination, presence of an undergraduate and postgraduate degree and place of radiology training were evaluated to assess the impact on the examination outcome of the candidate. Among other findings, the study demonstrated that those who trained within the UK performed better than those who trained outside the UK and that the FRCR 2B examination for UK candidates was unprejudiced with respect to gender and ethnicity.^8^ There is currently no local research on the pass rates and factors influencing the success of candidates training in South Africa.

On 23 March 2020, the president of South Africa declared a national lockdown because of the global coronavirus disease 2019 (COVID-19) pandemic.^9^ This had a great impact on service delivery in healthcare in the country. There was a disruption to personal and professional life as well as to the academic training of radiology registrars. Compliance with the pandemic restrictions, which included social distancing and infection control with the wearing of personal protective equipment (PPE), changed the dynamic of the general workflow.^10^ Staff shortages were the result of a reduction in the number of radiology registrars working at a particular time in order to reduce the spread of the virus in the workplace, which was further compounded by registrars who tested positive for the virus and were required to isolate.^10,11^ The shortage in the workforce and movement restrictions were particularly challenging for the University of the Witwatersrand (WITS) radiology circuit, where all registrars are required to rotate through various hospitals to gain clinical experience and exposure. Because of the restriction on movement, registrar rotations to these hospitals were completely stopped, and exposure to the different ‘special blocks’ and radiology modalities that included paediatrics, mammography, interventional radiology, CT, MRI, fluoroscopy and ultrasound, was limited. Furthermore, radiology registrars had to be redeployed to work in other clinical disciplines, mainly internal medicine, to assist with the crisis. The academic lectures that historically had always been ‘face to face’ were halted, along with multidisciplinary meetings with other specialities. This led to the introduction of online lectures and multidisciplinary online meetings, including frequent webinars hosted both locally and internationally. Overall, there was a negative impact on radiology training during this time. In line with social distancing practices, the pandemic led to a change in the examination structure, replacing the oral and handwritten components with a fully computer-based, typed examination.

The purpose of this study is to identify the factors affecting pass rates in the FC Rad Diag (SA) Part 2 examination and to explore the frequency of success. While the focus of this study is on the factors influencing examination outcomes, it is important to observe that the study period was interrupted by the COVID-19 pandemic. This disruption may have further impacted examination success rates, which will be considered in the analysis as an additional factor.

## Materials and methods

This was a descriptive, quantitative study that analysed the factors influencing the outcomes of the FC Rad Diag (SA) Part 2 examination. The study population consisted of all candidates who wrote the FC Rad Diag (SA) Part 2 examination during the period 01 January 2015–31 December 2023.

The database of the CMSA was accessed to retrieve statistics on factors influencing examination success and outcomes within the defined study period. A Microsoft Excel spreadsheet was used to capture the following data: demographic factors (age, marital status, post held at time of the examination); educational background (place of undergraduate training – whether in South Africa versus outside South Africa, the presence of a postgraduate qualification, and exemption to the CMSA Part I examination); examination-related factors (number of examination attempts, year of latest examination attempt, semester of sitting, and year of passing Part I).

### Data analysis and statistics

A statistical computing programming software, R version 3.6.3 of the R Core Team 2020, was used to produce descriptive and inferential statistics. Numerical variables were presented as five number summaries, including standard deviation and the coefficient of variation, depending on the distribution. Counts and percentage frequencies were used for summarising categorical variables. The numeric variables were further grouped; the mean differences and median were assessed using either the *t*-test or Wilcoxon test, depending on the distributional properties. The applicable tests for categorical variables were either a Chi-square test or Fisher’s exact test, depending on the cell count. The post hoc test for the categorical variable used a row-wise, paired *z*-test. A 5% level of significance was used as the benchmark to determine statistically significant results.

### Ethical considerations

The study was approved by the Human Research Ethics Committee (Medical) of the University of the Witwatersrand on 18 September 2023. The ethical clearance number is M201104. The CMSA Colleges of Medicine South Africa (CMSA) Management Committee approved both the study and access to the data. All candidate data were anonymised and allocated a random identifier number by the CMSA information specialist before the data were made available to the principal investigator to maintain confidentiality.

## Results

### Overall success rate and demographic factors

There were a total of 377 examination candidates during the study period, with an overall pass rate of 85.7% (*n* = 323, Chi-square *p*-value < 0.001). Candidates’ age had a significant impact on the examination outcome. Candidates who had not passed the examination were older than those who had passed (median age: 42 years old [37–47 years] vs. 35 years old [33–38 years], *p* < 0.001) (Table 1). There was no significant association between examination outcome and marital status (*p* = 0.251). The proportion of married candidates was similar between those who passed and those who had not yet passed (58.6% vs. 55.8%) (Table 1). Similarly, the proportion of single candidates was not significantly different between the two groups (38.1% vs. 40.4%).

**TABLE 1:** Demographic factors of candidates who passed and those who had not passed.

Exam outcome	Passed (*N* = 323)						Not yet passed (*N* = 54)						*P*	Overall (*N* = 377)					
	*n*	%	Median	IQR	*N*	Min–Max	*n*	%	Median	IQR	*N*	Min–Max		*n*	%	Median	IQR	*N*	Min–Max
**Age in years**																			
Median (Q1–Q3)	-	-	35.0	33.0–38.0	-	-	-	-	42.0	37.0–47.0	-	-	^$^*p* < 0.001	-	-	35.0	33.0–39.0	-	-
*N* (Min–Max)	-	-	-	-	287	30.0–50.0	-	-	-	-	51	32.0–56.0	-	-	-	-	-	338	30.0–56.0
**Marital status**	-	-	-	-	-	-	-	-	-	-	-	-	^#^*p* = 0.251	-	-	-	-	-	-
Married	177	58.6	-	-	-	-	29	55.8	-	-	-	-	-	206	58.2	-	-	-	-
Single	118	39.1	-	-	-	-	21	40.4	-	-	-	-	-	139	39.3	-	-	-	-
Divorced	7	2.3	-	-	-	-	1	1.9	-	-	-	-	-	8	2.3	-	-	-	-
Widowed	0	0.0	-	-	-	-	1	1.9	-	-	-	-	-	1	0.3	-	-	-	-
**Post at examination time**	-	-	-	-	-	-	-	-	-	-	-	-	^#^*p* < 0.001	-	-	-	-	-	-
Registrar	259	80.2	-	-	-	-	27	50.0	-	-	-	-	<0.001	286	75.9	-	-	-	-
Medical officer	64	19.8	-	-	-	-	27	50.0	-	-	-	-	<0.001	91	24.1	-	-	-	-
**Specific category of training post at examination time**	-	-	-	-	-	-	-	-	-	-	-	-	^#^*p* < 0.001	-	-	-	-	-	-
South African citizen or permanent resident registrar	221	68.4	-	-	-	-	24	44.4	-	-	-	-	0.002	245	65.0	-	-	-	-
Supernumerary registrar	38	11.8	-	-	-	-	3	5.6	-	-	-	-	0.238	41	10.9	-	-	-	-
Medical officer	64	19.8	-	-	-	-	27	50.0	-	-	-	-	< 0.001	91	24.1	-	-	-	-

*Source:* Authors’ analysis of Colleges of Medicine of South Africa (CMSA) examination data (2015–2023)

Age data were available for 338 participants (39 missing) and marital status data for 354 participants (23 missing). Percentages were calculated using available responses for each variable.

# Fisher’s exact/Chi-square test;

$ RankSum test; Wilcoxon.

There was a significant association between examination outcome and the candidate’s post at the time of the examination (Table 1 and Table 2). Medical officers were candidates who were no longer part of the formal registrar programme but had completed their formal registrar training time; however, they had not passed their final examination within the registrar programme. There was a higher chance of passing the examination within a formal training post (as a registrar) compared to being outside the formal training post (as a medical officer), with a high proportion of failures among medical officers (50%). There was no statistical significance in examination outcome between South African or permanent registrars compared to their supernumerary counterparts (Table 1).

**TABLE 2:** The effect of the type of post held by candidates on examination outcomes.

Specific category of training post at time of examination	South African or permanent resident registrar: supernumerary registrar	South African or permanent resident registrar: medical officer	Supernumerary registrar: medical officer

Pairwise total	*n* = 286	*n* = 336	*n* = 132
**Exam outcome ^#^*p* < 0.001**			
Passed	1.000	< 0.001	0.007
Not yet passed	1.000	< 0.001	0.007

*Source:* Authors’ analysis of Colleges of Medicine of South Africa (CMSA) examination data (2015–2023)

# Chi-square test.

### Educational factors

There was a notable association between the number of years post MBBCH or MBChB and examination outcome. The median number of years since MBBCH or MBChB for candidates who passed was 11.0 years, and was lower when compared to candidates who had not passed; 14.5 years, *p* < 0.001 (Table 3). The presence of a post-graduate qualification was defined by what was declared on the application form. The majority of candidates (n = 326/377, 86.5%) did not have a post-graduate qualification. The presence of a postgraduate qualification was unrelated to the examination outcome (*p* = 0.247) (Table 1). Exemption from writing the CMSA Part 1 examination did not influence success in the Part 2 examination (*p* = 0.802) (Table 4).

**TABLE 3:** The association between the Colleges of Medicine of South Africa Part 2 examination attempts and examination outcome.

Examination outcome	Passed (*N* = 323)						Not yet passed (*N* = 54)						*P*	Overall (*N* = 377)					
	*n*	%	Median	IQR	*N*	Min–Max	*n*	%	Median	IQR	*N*	Min–Max		*n*	%	Median	IQR	*N*	Min–Max
**Count of attempts**	-	-	-	-	-	-	-	-	-	-	-	-	^#^*p* < 0.001	-	-	-	-	-	-
1	236	73.1	-	-	-	-	7	13.0	-	-	-	-	< 0.001	243	64.5	-	-	-	-
2	47	14.6	-	-	-	-	12	22.2	-	-	-	-	0.429	59	15.6	-	-	-	-
3	19	5.9	-	-	-	-	8	14.8	-	-	-	-	0.196	27	7.2	-	-	-	-
4	10	3.1	-	-	-	-	10	18.5	-	-	-	-	< 0.001	20	5.3	-	-	-	-
5	2	0.6	-	-	-	-	6	11.1	-	-	-	-	0.001	8	2.1	-	-	-	-
6	6	1.9	-	-	-	-	5	9.3	-	-	-	-	0.071	11	2.9	-	-	-	-
7	1	0.3	-	-	-	-	2	3.7	-	-	-	-	0.220	3	0.8	-	-	-	-
8	1	0.3	-	-	-	-	0	0.0	-	-	-	-	1.000	1	0.3	-	-	-	-
9	1	0.3	-	-	-	-	3	5.6	-	-	-	-	0.071	4	1.1	-	-	-	-
13	0	0.0	-	-	-	-	1	1.9	-	-	-	-	0.429	1	0.3	-	-	-	-
**Number of attempts**	-	-	-	-	-	-	-	-	-	-	-	-	-	-	-	-	-	-	-
Median (Q1–Q3)	-	-	1.00	1.00–2.00	-	-	-	-	3.50	2.00–5.00	-	-	^$^< 0.001	-	-	1.00	1.00–2.00	-	-
*N* (Min–Max)	-	-	-	-	323	1.00–9.00	-	-	-	-	54	1.00–13.0	-	-	-	-	-	377	1.00–13.0
**Examination attempts**	-	-	-	-	-	-	-	-	-	-	-	-	^#^*p* < 0.001	-	-	-	-	-	-
Single	236	73.1	-	-	-	-	7	13.0	-	-	-	-	< 0.001	243	64.5	-	-	-	-
Multiple	87	26.9	-	-	-	-	47	87.0	-	-	-	-	< 0.001	134	35.5	-	-	-	-
**Attempts**	-	-	-	-	-	-	-	-	-	-	-	-	^#^*p* < 0.001	-	-	-	-	-	-
1	236	73.1	-	-	-	-	7	13.0	-	-	-	-	< 0.001	243	64.5	-	-	-	-
2	47	14.6	-	-	-	-	12	22.2	-	-	-	-	0.159	59	15.6	-	-	-	-
≥ 3s	40	12.4	-	-	-	-	35	64.8	-	-	-	-	< 0.001	75	19.9	-	-	-	-
**Time from MBBCH to Part 2 latest attempt**	-	-	-	-	-	-	-	-	-	-	-	-	-	-	-	-	-	-	-
Median (Q1–Q3)	-	-	11.0	9.00–13.0	-	-	-	-	14.5	12.0–17.0	-	-	^$^< 0.001	-	11.0	-	9.00–13.0	-	-
*N* (Min–Max)	-	-	-	-	322	7.00–23.0	-	-	-	-	54	9.00–30.0	-	-	-	-	-	376	7.00–30.0
**Time from Part 1 to Part 2 latest attempt**	-	-	-	-	-	-	-	-	-	-	-	-	-	-	-	-	-	-	-
Median (Q1–Q3)	-	-	4.00	3.00–5.00	-	-	-	-	5.00	4.00–6.00	-	-	^$^< 0.001	-	-	4.00	4.00–5.00	-	-
*N* (Min–Max)	-	-	-	-	323	0–8.00	-	-	-	-	54	2.00–9.00	-	-	-	-	-	377	0–9.00

*Source:* Authors’ analysis of Colleges of Medicine of South Africa (CMSA) examination data (2015–2023)

MBBCH, Bachelor of Medicine.

# Fisher’s exact/Chi-Square test;

$ RankSum test; Wilcoxon.

**TABLE 4:** Educational factors affecting examination outcomes.

Exam outcome	Passed (*N* = 323)		Not yet passed (*N* = 54)		*P*	Overall (*N* = 377)	
	*n*	%	*n*	%		*n*	%
**Undergraduate training location**	-	-	-	-	^#^*p* = 0.994	-	-
SA	287	88.9	48	88.9	-	335	88.9
Non-SA	36	11.1	6	11.1	-	42	11.1
**Postgraduate qualification**	-	-	-	-	^#^*p* = 0.247	-	-
No	282	87.3	44	81.5	-	326	86.5
Yes	41	12.7	10	18.5	-	51	13.5
**Exempt from CMSA Part I**	-	-	-	-	^#^*p* = 0.802	-	-
No	285	88.2	47	87.0	-	332	88.1
Yes	38	11.8	7	13.0	-	45	11.9

*Source:* Authors’ analysis of Colleges of Medicine of South Africa (CMSA) examination data (2015–2023)

CMSA, Colleges of Medicine of South Africa; SA, South Africa.

# Fisher’s exact/Chi-square test.

Candidates received an exemption from writing the Part 1 examination when the CMSA had reciprocity with another institution. The proportion of those who did not receive CMSA Part 1 exemption was not significantly different between those who passed and had not yet passed (88.2% vs. 87.0%) (Table 4). The location of undergraduate medical training, whether in South Africa or outside South Africa, was not significantly associated with examination success (Table 4).

### Examination-related factors

An examination written in the first or second semester had no bearing on the examination outcome (*p* = 0.542). However, there was a trend towards a higher pass rate in the first semester (Table 5). There was a notable association between the year in which the examination was written and the outcome (*p* < 0.001; Table 5). Higher pass rates were recorded before 2020 (53.3%) compared to 2020 and beyond (46.7%) (*p* = 0.004).

**TABLE 5:** Examination factors associated with examination outcome.

Exam outcome	Passed (*N* = 323)		Not yet passed (*N* = 54)		*P*	Overall (*N* = 377)	
	*n*	%	*n*	%		*n*	%
**Semester sitting part II**	-	-	-	-	^#^*p* = 0.542	-	-
First semester	170	52.6	26	48.1	-	196	52.0
Second semester	153	47.4	28	51.9	-	181	48.0
**Examination period**	-	-	-	-	^#^*p* = 0.001	-	-
Before 2020	172	53.3	16	29.6	0.004	188	49.9
2020 and beyond	151	46.7	38	70.4	0.004	189	50.1

*Source:* Authors’ analysis of Colleges of Medicine of South Africa (CMSA) examination data (2015–2023)

# Fisher’s exact/Chi-square test.

Among all candidates who failed the examination, a significantly larger proportion did so between 2020 and 2023 (70.4%) compared to before 2020 (29.6%) (Table 5). The number of candidates who passed had a shorter time between Part 1 and Part 2; the median number of years was 4 years versus 5 years for those who had not passed (*p* < 0.001, Table 3).

The majority of candidates (73.1%) passed on their first attempt (Table 4). A significant number of candidates (18.5%), who had not yet passed, had attempted the examination at least four times. The highest number of attempts recorded was 13, and this candidate has not yet passed. There was a higher proportion of registrars who passed on the first attempt compared to medical officers (74.8% vs. 31.9%) (Table 6). Multiple attempts at the examination were made by medical officers (68.1%) compared to registrars (25.2%) (Table 6).

**TABLE 6:** The relationship between the number of Colleges of Medicine of South Africa Part 2 examination attempts and the type of post held by candidates.

Post at examination time	Registrar (*N* = 286)		Medical officer (*N* = 91)		*P* ^#^	Overall (*N* = 377)	
	*n*	%	*n*	%		*n*	%
**Count of attempts**	-	-	-	-	*p* < 0.001	-	-
1	214	74.8	29	31.9	< 0.001	243	64.5
2	43	15.0	16	17.6	1.000	59	15.6
3	17	5.9	10	11.0	0.500	27	7.2
4	5	1.7	15	16.5	< 0.001	20	5.3
5	4	1.4	4	4.4	0.500	8	2.1
6	1	0.3	10	11.0	< 0.001	11	2.9
7	0	0.0	3	3.3	0.082	3	0.8
8	1	0.3	0	0.0	1.000	1	0.3
9	0	0.0	4	4.4	0.023	4	1.1
13	1	0.3	0	0.0	1.000	1	0.3
**Exam attempts**	-	-	-	-	*p* < 0.001	-	-
Single	214	74.8	29	31.9	< 0.001	243	64.5
Multiple	72	25.2	62	68.1	< 0.001	134	35.5
**Attempts**	-	-	-	-	*p* < 0.001	-	-
1	214	74.8	29	31.9	< 0.001	243	64.5
2	43	15.0	16	17.6	0.619	59	15.6
≥ 3	29	10.1	46	50.5	< 0.001	75	19.9

*Source:* Authors’ analysis of Colleges of Medicine of South Africa (CMSA) examination data (2015–2023)

# Fishers exact/Chi-square test.

## Discussion

The aim of this study was to determine the overall pass rate and factors affecting examination outcome for the FC Rad (SA) Part 2. The observed overall pass rate of 85.7% was higher than that reported for the FRCR Part 2B (UK) at 59.4%.^8^ It is possible that variations in pass rates resulted from different study periods between this study and the UK study, which described data for the 2006–2010 period. Changes to the examination formats in the FC Rad Diag (SA) Part 2 were also implemented in the 2010–2011 period, including introducing a digital platform for long cases and rapid reporting, which might have had an impact on pass rates.^12^ Decreased pass rates have been linked to factors such as gender, ethnicity, and non-UK place of radiology training in the UK study.^8^ This study found that younger age, post at time of the examination, pre-2020 examinations, passing at first attempt, and less time elapsed between MBBCH or MBChB and the Part 1 to Part 2 examinations, had a positive association with the examination outcome.

Age was found to play a role in the outcomes of the Part 2 examination, with younger candidates passing the Part 2 examination more frequently than older candidates. This outcome was similar to the findings in a study by Gao et al. in China, which showed that one of the factors that negatively influenced examination outcomes was advanced age. It was postulated that older candidates would have difficulty adjusting to new standardised training requirements after having had clinical work experience and developed personal work habits.^6^ Their study also observed that candidates with a lower number of years after completing medical school were more likely to pass the examinations.^6^ The present study is comparable, also noting that candidates with a shorter time period between medical school and Part 2 examinations were more likely to pass. A shorter gap between foundational medical training and specialisation may be advantageous, potentially because of better retention of core medical knowledge, continued academic momentum and stronger study habits.^6^

Medical officers had lower pass rates and more examination attempts compared to registrars, including supernumerary registrars. Supernumerary registrars are sponsored, unpaid registrars or non-South African registrars without permanent resident status in the Republic of South Africa; the majority originate from African countries with limited or no access to specialist training.^11^ One explanation for lower medical officer pass rates could be that medical officers attempting the examinations are no longer part of the formal registrar training programme and may lack adequate supervision and access to teaching resources such as examination-focused tutorials and practice examination material. This is possible because most medical officers, after completing their registrar time, are required to vacate their registrar post for the incoming new registrars. This often means leaving the academically accredited training hospitals to seek employment as a medical officer in smaller regional hospitals without a formal, academically inclined radiology training programme. In contrast, registrars and supernumerary registrars work within supervised training programmes where mistakes and gaps in knowledge can be identified and rectified.^13^ Inadequate supervision may contribute to increased stress and reduced motivation, both of which are associated with poorer examination performance.^14^ Furthermore, medical officers are less exposed to the daily academic rigours of tutorials, case presentations, internal department assessments and multidisciplinary meetings that are aimed at preparing the candidate to pass the examination. Registrars were therefore more likely to be better prepared for the examination than their medical officer counterparts.

Failure to pass the examinations on the first attempt was a significant risk factor for subsequent failures in the FC Rad (SA) Part 2 examination. This finding was concordant with a similar study in the UK on the FRCR Part 2B examination, where a reduction in pass rates was found with an increasing number of attempts.^8^ Multiple unsuccessful examination attempts add a significant mental burden and stress, as career progression is on hold until success in the examination is achieved, which can adversely affect performance.^14,15^

The COVID-19 pandemic had an impact on radiology training with changes in the workflow, academic programme and examination format.^16,17^ This study showed a significant increase in failure rates recorded from the year 2020 and onwards, after the COVID-19 national lockdown was declared in the country. The change in examination structure from written and oral examinations to a completely computer- based typed exam was likely a contributing factor. A study performed in 2016 on the *Review of FC Rad Diag SA examination results over ten years* explored the changing examination format and its effect on the examination results.^12^ The study showed a significant reduction in the FC Rad (SA) Part 2 pass rates when the examination format had been changed to include a digital platform, among other changes.^12^ Possible explanations include candidates’ unfamiliarity with the digital software and technical issues affecting candidate performance. Simulated assessments in the new format could help candidates become more familiar with the structure and improve examination preparation. A study by Veerasuri et al. confirmed that radiology workload decreased in response to COVID-19, with decreased subspecialty experience and a lack of subspecialty training.^16^ The reduction in adequate training is likely another contributing factor to increasing failure rates since 2020.^18,19^ Furthermore, the negative psychological impact of the COVID-19 pandemic on candidate performance likely played a role in the examination outcome.^10,20^

### Study’s limitations

Some of the participants assumed different posts over the years when they attempted examinations, so a standard reference point could only be determined using the post at the latest attempt. Marital status is likely to have changed over the years with no information provided on which year the marital status was attained.

Candidates did not necessarily complete their examination attempts within the same semester. To standardise the analysis, each candidate was classified according to the semester in which their most recent attempt was made.

### Recommendations

Efforts must be made to prepare registrars for the new examination format. Candidates must be encouraged and supported to write and pass the Part 2 examinations within the registrar programme, as the study finds that the chances of passing are much higher within formal registrar training posts. Candidates in medical officer posts should be surveyed to determine the challenges experienced that forced them to sit for examinations only after completing their registrar time, and the factors resulting in multiple examination attempts. Training institutions should identify candidates who require extra support or remediation and assist them in their examination preparation. A follow-up study in 5 years is recommended to assess any recovery in the post-COVID-19 pass rates. Furthermore, candidate questionnaires can be considered to explore the challenges faced with the post-COVID-19 new examination format.

## Conclusion

The analysis of the FC Rad Diag (SA) Part 2 examination data suggests that although pass rates are high, the factors that influence success are multifactorial. Candidates are encouraged to write and pass the Part 2 examinations within the registrar programme, as this study finds that the chances of passing are much higher within formal registrar training posts. Candidates are encouraged to pass on their first attempt, as there is a high risk of subsequent failures after a single attempt. The COVID-19 pandemic, with the change in examination structure, had a significant impact, with a reduction in the pass rates. These insights may inform candidate preparation strategies and guide training institutions in supporting examination success.
